# Determinants of vessel defects in superficial and deep vascular layers in normal-tension glaucoma using optical coherence tomography angiography

**DOI:** 10.1038/s41598-021-89428-5

**Published:** 2021-05-11

**Authors:** Jiyun Lee, Chan Kee Park, Hae-Young Lopilly Park

**Affiliations:** Department of Ophthalmology, Seoul St. Mary’s Hospital, College of Medicine, The Catholic University of Korea, 222, Banpo-daero, Seocho-gu, 06591 Seoul, Republic of Korea

**Keywords:** Optic nerve diseases, Glaucoma

## Abstract

We investigated the characteristics of localized vessel density defects (VD) either in the deep or superficial vascular layer of normal-tension glaucoma patients using optical coherence tomography angiography (OCTA). In this retrospective, cross-sectional study, 74 eyes with localized retinal nerve fiber layer (RNFL) defect were included. The relationships between the widths of the VD in the superficial and deep layer and ocular factors were evaluated. Eyes with greater deep VD were significantly older (*P* = 0.023). The IOP measured at OCTA exam was significantly related to the width of the deep VD (*P* = 0.009). By contrast, average ganglion cell inner plexiform layer thickness (GCIPLT) was substantially related to the width of the superficial VD (*P* = 0.004). In logistic regression analysis, aging was noticeably associated with wider deep VD, whereas worse mean deviation (MD) had a significant association with wider superficial VD (*P* = 0.001, *P* = 0.003, respectively). In sum, while changes of the superficial layer seemed an overall ramification of glaucomatous damages, the deep layer was more likely to be affected by factors related to ocular microcirculation, such as IOPs and older age. Thus, looking into the deep vascular layer using OCTA could identify risk factors related to the disturbance in ocular microcirculation.

## Introduction

Glaucoma, a progressive optic neuropathy, is a result of irreversible but continuous loss of retinal ganglion cells and their axons due to mechanical stresses on lamina cribrosa^1^. Generally, intraocular pressure (IOP) has been regarded one of the major risk factors for the mechanical stress, however, large, randomized clinical studies such as the Collaborative Normal Tension Glaucoma Study (CNTG) and the Early Manifest Glaucoma Trial (EMGT) showed that migraine, disc hemorrhages, or lower ocular systolic perfusion pressure affected substantially on progression of the disease^2,3^. Furthermore, precedent studies using fluorescein angiography found hypoperfusion in optic disc of glaucoma patients and suggested that their optic disc might have susceptible vasculature regardless of IOP elevation^4,5^. Other studies using color doppler imaging system found that decreased or impaired in retinal microcirculation and retrobulbar blood flow might be associated with glaucoma^6,7^.

Lately, optical coherence tomography angiography (OCTA) detecting blood flow through the motion contrast generated by red blood cells has been developed, and has been a useful implement for accessing microcirculation in peripapillary, macular, and choroid layers. According to studies via OCTA, blood circulation in various layers including optic nerve head^8,9^, superficial peripapillary^9,10^, macular^11^, and foveal avascular zone area^12^ was associated with severity of glaucoma. Nonetheless, superficial layers were only included in previous studies, and this might be due to the fact that superficial retinal nerve fiber layer (RNFL) and ganglion cell layer (GCL) are main sites experiencing glaucomatous damage, and therefore, the microcirculation of the deep layer might be less likely to be thwarted. In addition, deep layer of OCTA images might be hindered due to projection artefacts (PA)^13^. However, with the development of the PA removal software, the deep layer of OCTA images became involved in the evaluation^14,15^.

In terms of changes in the deep vascular layer, Jeon et al. found that deep macular vessel density was an independent risk factor of central visual function and retinal thickness thinning^16^, and their subsequent study suggested that deep macular vessel density might be able to be a predictor of glaucomatous visual field (VF) progression related with vascular incompetence^17^.

Considering the fact that deep layer of the OCTA might reflect changes of microcirculation caused by glaucoma, it may be important to look into related factors to OCTA changes in both superficial and deep layers. Therefore, the aim of this study was to find out associated factors to VD at the site of localized RNFL defect in both superficial and deep layers and compare the features between patients with wider VD in the superficial or deep layers on OCTA.

## Results

Initially, 194 eyes were recruited, however, eyes were excluded: 74 eyes due to less than 10° difference between the two layers, 33 eyes due to lack of presence of the VDs in the both layers, and 15 eyes whose image quality score less than 50. The rest of 72 eyes of 72 patients with NTG who satisfied the inclusion and exclusion criteria were included for further study.

Patient’s characteristics are in Table 1. The widths for the superficial and the deep layers of the SVD group were 46.56 ± 21.86°, and 32.16 ± 17.20°, respectively. Those for the DVD group were 35.11 ± 18.83°, and 59.97 ± 24.87°, respectively. The DVD group was significantly older than the SVD group (*P*, 0.023). Regarding the degree of visual field defects, the SVD group showed substantially progressed status not only in mean deviations (MD) but also in pattern standard deviation (PSD), and visual field index (VFI) (all Ps ≤ 0.002). In addition, the DVD group showed a higher proportion of the paracentral VF defect, compared to the SVD group (% of paracentral VF defects, 47.6%, 28.1%, respectively, *P* 0.008).

**Table 1:** Patient’s demographics.

	Wider SVD (N,32)	Wider DVD (N,42)	*P* value
OCTA SSI	67.34 ± 5.42	64.98 ± 7.21	0.127^a^
Width of Superficial defects (°)	46.56 ± 21.86	35.11 ± 18.83	**0.018**^a^
Width of deep defects (°)	32.16 ± 17.20	59.97 ± 24.87	** < 0.001**^a^
Age (year)	52.06 ± 11.99	58.48 ± 11.63	**0.023**^a^
Sex (Male, no. (%))	10 (31.3%)	18 (42.9%)	0.308^b^
Laterality (OD, no. (%))	19 (59.4%)	16 (38.1%)	*0.069*^b^
HTN (Yes, no. (%))	2 (6.3%)	9 (21.4%)	0.101^b^
DM (Yes, no. (%))	2 (6.3%)	3 (7.1%)	1.000^b^
Cardiovascular disease (Yes, no. (%))	1 (3.1%)	4 (9.5%)	0.639^b^
Migraine (Yes, no. (%))	1 (3.1%)	3 (7.1%)	0.629^b^
Cold extremities (Yes, no. (%))	1 (3.1%)	4 (9.5%)	0.639^b^
Baseline IOP (mmHg)	15.23 ± 3.95	15.40 ± 3.31	0.834^a^
IOP (mmHg) measured at OCTA exam	13.0 ± 2.72	14.31 ± 2.93	0.054^a^
CCT (µm)	529.70 ± 45.70	527.71 ± 32.29	0.830^a^
AL (mm)	24.51 ± 5.23	25.06 ± 1.57	0.540^a^
MD (dB)	− 4.71 ± 3.05	− 2.52 ± 2.35	**0.001**^a^
PSD (dB)	6.66 ± 3.72	3.72 ± 2.85	**0.001**^a^
VFI (%)	88.16 ± 9.42	94.67 ± 6.11	**0.002**^a^
Presence of Paracentral VFD (Yes, no. (%))	9 (28.1%)	20 (47.6%)	**0.008**^b^
Average RNFLT (µm)	73.25 ± 9.20	76.62 ± 8.75	0.113^a^
Average CD ratio	.72 ± .11	.69 ± .15	0.334^a^
Average GCIPLT (µm)	67.41 ± 8.05	70.05 ± 6.99	0.136^a^

*SVD* superficial vessel defect, *DVD* deep vessel defect, n = number, *OCTA* optical coherence tomography angiography, *SSI* signal strength index, *HTN* hypertension, *DM* diabetes mellitus, *IOP* intraocular pressure, *CCT* central corneal thickness, *AL* axial length, *MD* mean deviation, *dB* decibel, *PSD* pattern standard deviation, *VFI* visual field index, *VFD* visual field defect, *RNFLT* retinal nerve fiber layer thickness, *CD* cup disc, *GCIPLT* ganglion cell inner plexiform layer thickness.

Mean values are presented with standard deviations.

Bold font indicates significant *P* values (*P* < 0.05).

^a^Student’s t-test.

^b^Chi-squared test.

With regard to the OCT parameters (Table 1), there were no noticeable differences in average RNFL thickness (RNFLT), average cup-to-disc (C/D) ratio, average ganglion cell inner plexiform layer thickness (GCIPLT).

**Figure 1:**
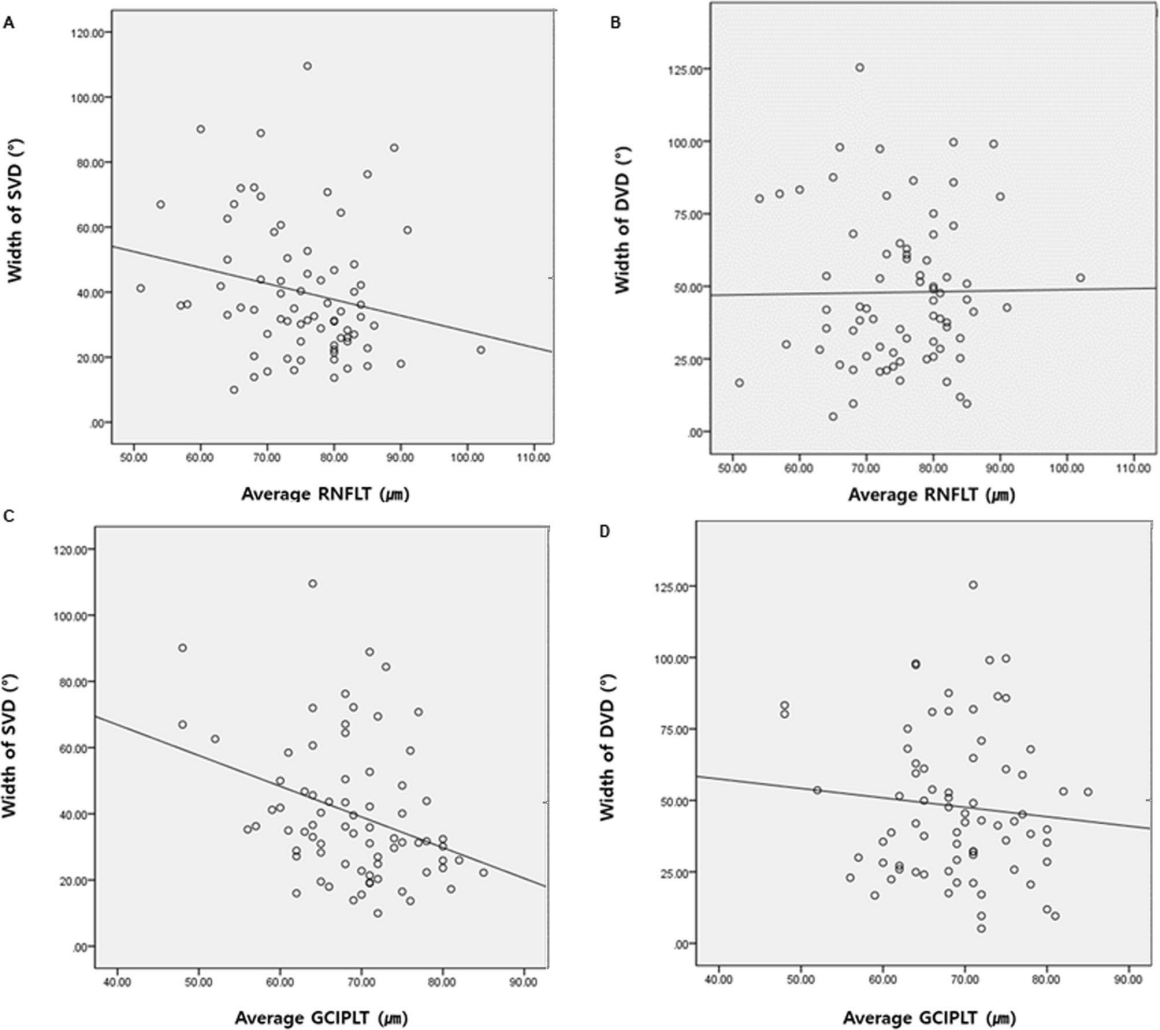
Relationship between the width of vessel density defect in superficial or deep layers and structural parameters, including average retinal nerve fiber layer thickness and average ganglion cell inner plexiform layer thickness. The correlation between the width of superficial vessel defect (SVD) and the average retinal nerve fiber layer thickness (RNFLT) (**A**) and average ganglion cell inner plexiform layer thickness (GCIPLT) (**C**) was substantially significant, compared to the correlation between the width of deep vessel defect (DVD) and average RNFLT (**B**) and average GCIPLT (**D**). Pearson correlation analysis was used.

**Figure 2:**
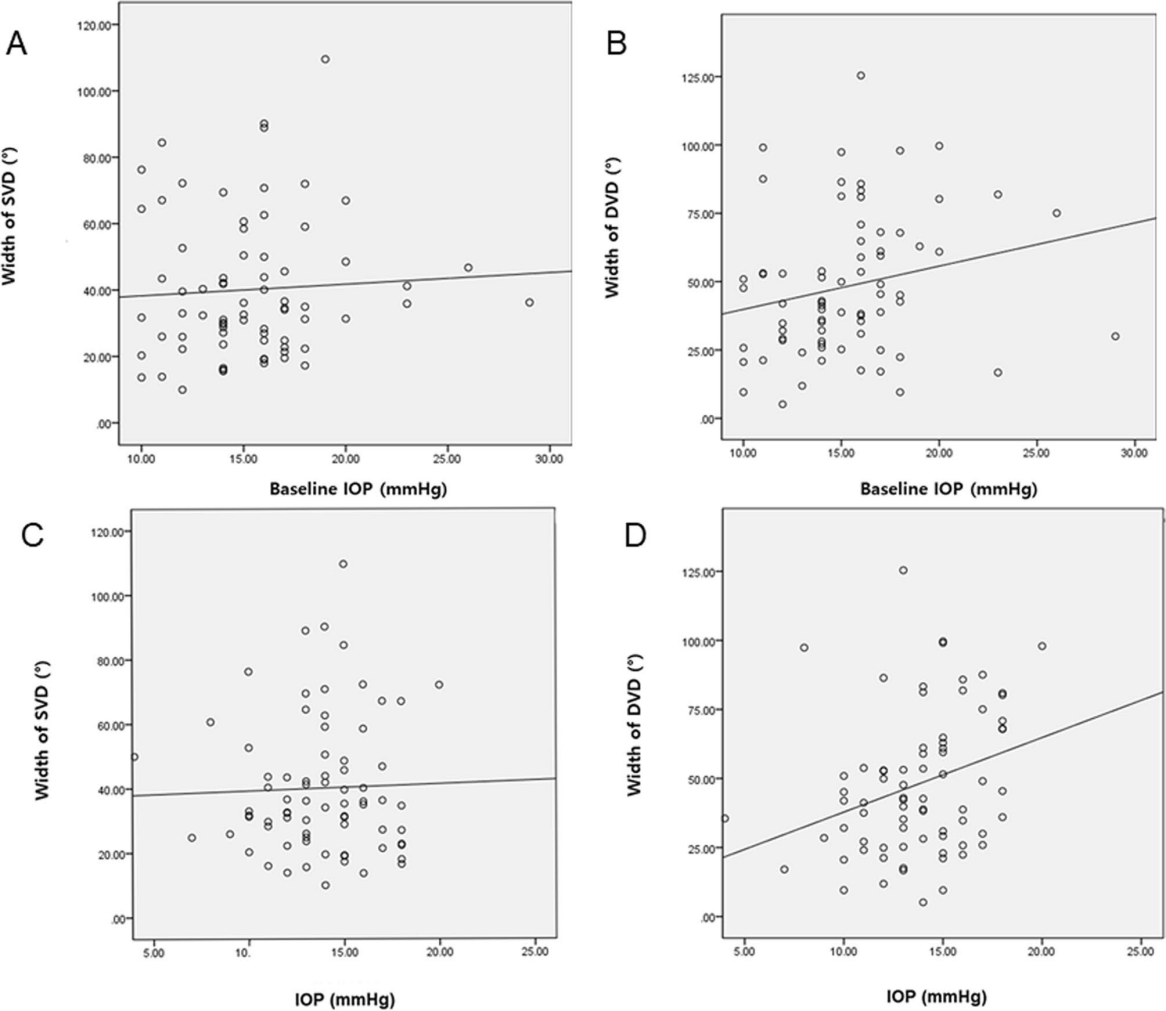
Relationship between the Width of Vessel Density Defect in Superficial or Deep layers and Baseline Intraocular Pressure, measured Intraocular Pressure at OCTA examination. The correlation between the width of superficial vessel defect (SVD) and baseline intraocular pressure (IOP) (**A**) and the IOP measured at OCTA examination (**C**) did not show statistical significance. Nevertheless, the width of deep vessel defect (DVD) presented markedly significant relationships with the baseline and the measured intraocular pressures (**B**,**D**). Pearson correlation analysis was used.

The Pearson correlation analysis was performed between clinical parameters and the width of the VDs in the superficial and the deep layers in the total eyes (Table 2). The average GCIPLT showed a marked correlation with the widths of the VD in the superficial layer (r, − 0.335, *P*, 0.004). MD and average RNFLT showed borderline correlation with the width of the superficial VD (r, − 0.226, *P*, 0.054, r, − 0.213, *P*, 0.068, respectively). Meanwhile, the IOP measured at the OCTA exam showed a significant correlation with the width of the deep VD (r, 0.303, *P*, 0.009), and baseline IOP was marginally correlated with the width of the deep VD (r, 0.219, *P*, 0.063). In Fig. 1, the width of the superficial VD increases as average RNFLT and average GCIPLT decreases (R^2^, 0.045, *P*, 0.068, R^2^, 0.112, *P*, 0.04, respectively), yet these relationships were attenuated with the width of the deep VD (R^2^, 0.048, *P*, 0.914, R^2^, 0.0002, *P*, 0.415, respectively). Nonetheless, the width of deep VD increases as baseline and measured IOP increases (Fig. 2). These relationships between the IOPs and the width of deep VD were not found with the width of superficial VD. (R^2^, 0.048, *P*, 0.063, R^2^, 0.004, *P*, 0.616, respectively, for baseline IOP, R^2^, 0.092, *P*, 0.009, R^2^, 0.001, *P*, 0.798, respectively, for the IOP measured at OCTA exam).

**Table 2:** Correlation coefficients between the width of vessel defect in each layer and structural or functional parameters in all eyes.

	Width of SVD	*P*	Width of DVD	*P*
Age	− 0.097	0.410	0.162	0.167
Baseline IOP	0.060	0.616	0.219	0.063
IOP measured at OCTA exam	0.030	0.798	0.303	**0.009**
CCT	− 0.023	0.852	0.065	0.593
Axial Length	0.042	0.737	0.038	0.764
MD	− 0.226	0.054	0.096	0.418
PSD	0.092	0.439	− 0.166	0.160
VFI	− 0.187	0.112	0.108	0.365
Average RNFLT	− 0.213	0.068	0.013	0.914
Average CD ratio	0.159	0.177	0.065	0.583
Average GCIPLT	− 0.335	**0.004**	− 0.096	0.415

*SVD* superficial vessel defect, *DVD* deep vessel defect, *IOP* intraocular pressure, *OCTA* optical coherence tomography angiography, *CCT* central corneal thickness, *MD* mean deviation, *PSD* pattern standard deviation, *VFI* visual function index, *RNFLT* retinal nerve fiber layer thickness, *CD* cup to disc, *GCIPLT* ganglion cell inner plexiform layer thickness.

Bold font indicates significant *P* values (*P* < 0.05).

Pearson correlation analysis was used.

Further analyses were carried out with patients whose MDs were worse than − 3 dB and better than − 6 dB (Table 3). The average GCIPLT was the only factor which correlated with the width of the superficial VD (r, − 0.325, *P*, 0.041), while the IOP factors including baseline and measured IOP, and central corneal thickness (CCT) showed significant correlations with the width of the deep VD (r, 0.0447, *P*, 0.004, r, 0.405, *P*, 0.010, and r, 0.377, *P*, 0.018, respectively).

**Table 3:** Correlation coefficients between the width of vessel defect in each layer and structural or functional parameters in eyes with MD <  − 3 dB.

	Width of SVD	*P*	Width of DVD	*P*
Age	− 0.088	0.591	0.288	0.071
Baseline IOP	− 0.060	0.715	0.447	**0.004**
IOP measured at OCTA exam	0.118	0.469	0.405	**0.010**
CCT	0.024	0.886	0.377	**0.018**
Axial Length	− 0.048	0.783	0.054	0.755
MD	− 0.158	0.330	0.086	0.596
PSD	− 0.003	0.988	− 0.058	0.724
VFI	− 0.041	0.800	0.137	0.399
Average RNFLT	− 0.240	0.136	− 0.031	0.851
Average CD ratio	0.007	0.964	0.002	0.990
Average GCIPLT	− 0.325	**0.041**	0.061	0.709

*SVD* superficial vessel defect, *DVD* deep vessel defect, *IOP* intraocular pressure, *OCTA* optical coherence tomography angiography, *CCT* central corneal thickness, *MD* mean deviation, *PSD* pattern standard deviation, *VFI* visual function index, *RNFLT* retinal nerve fiber layer thickness, *CD* cup to disc, *GCIPLT* ganglion cell inner plexiform layer thickness.

Bold font indicates significant *P* values (*P* < 0.05).

Pearson correlation analysis was used.

Linear regression analysis was adopted to identify factors associated with the width of VD in each layer (Table 4). Both baseline and measured IOPs were associated with the width of the deep VD (β, 1.582, *P*, 0.063, β, 2.699, *P*, 0.009, respectively) in the univariate analysis, however, in the multivariate analysis, only the measured IOP showed a significant association (β, 2.758, *P*, 0.007). As for the width of the superficial VD (Table 5), structural factors including average RNFLT and average GCIPLT and MD, a functional component, were associated with the width of the superficial VD. Nevertheless, in the multivariate analysis, average GCIPLT turned out to be the sole factor.

**Table 4:** Linear regression analysis to determine the correlation between variables and width of deep vascular defect in all eyes (N, 72).

	Univariate		Multivariate	
	Beta	*P*	Beta	*P*
Age	0.345	0.167		
Baseline IOP	1.582	**0.063**		
IOP (mmHg) measured at OCTA exam	2.699	**0.009**	2.758	**0.007**
CCT	0.044	0.593		
Axial Length	0.282	0.764		
MD	0.860	0.418		
PSD	− 1.203	0.160		
VFI	0.333	0.365		
Average RNFLT	0.036	0.914		
Average CD ratio	12.369	0.583		
Average GCIPLT	− 0.330	0.415		

*SVD* superficial vessel defect, *DVD* deep vessel defect, *IOP* intraocular pressure, *OCTA* optical coherence tomography angiography, *CCT* central corneal thickness, *MD* mean deviation, *PSD* pattern standard deviation, *VFI* visual function index, *RNFLT* retinal nerve fiber layer thickness, *CD* cup to disc, *GCIPLT* ganglion cell inner plexiform layer thickness.

Only variables with a *P* value < 0.10 in the univariate analysis were included in the multivariate model.

Bold font indicates significant *P* values (*P* < 0.05).

**Table 5:** Linear regression analysis to determine the correlation between variables and width of superficial vascular defect in all eyes (N, 72).

	Univariate		Multivariate	
	Beta	*P*	Beta	*P*
Age	− 0.167	0.410		
Baseline IOP	0.351	0.616		
IOP measured at OCTA exam	0.217	0.798		
CCT	− 0.013	0.852		
Axial Length	0.251	0.737		
MD	− 1.653	**0.054**		
PSD	0.544	0.439		
VFI	− 0.475	0.112		
Average RNFLT	− 0.492	**0.068**		
Average CD ratio	24.427	0.177		
Average GCIPLT	− 0.928	**0.004**	− 0.935	**0.004**

*SVD* superficial vessel defect, *DVD* deep vessel defect, *IOP* intraocular pressure, *OCTA* optical coherence tomography angiography, *CCT* central corneal thickness, *MD* mean deviation, *PSD* pattern standard deviation, *VFI* visual function index, *RNFLT* retinal nerve fiber layer thickness, *CD* cup to disc, *GCIPLT* ganglion cell inner plexiform layer thickness.

Only variables with a *P* value < 0.10 in the univariate analysis were included in the multivariate model.

Bold font indicates significant *P* values (*P* < 0.05).

Table 6 illustrates the results of logistic regression analysis to identify factors related to presence of wider DVD. In multivariate analysis, older age and MD showed significance (Both Ps, 0.001).

**Table 6:** Logistic regression analysis of factors associated with the presence of wider deep vascular defect.

	Univariate		Multivariate	
	β (95% CI)	*P* value	β (95% CI)	*P* value
Age (year)	0.046 (1.005–1.091)	**0.027**	0.047 (1.001–1.097)	**0.001**
Baseline IOP (mmHg)	0.014 (0.889–1.157)	0.831		
IOP measured at OCTA exam (mmHg)	0.166 (0.993–1.403)	**0.060**		
CCT (µm)	− 0.001 (0.986–1.011)	0.827		
Axial Length (mm)	0.045 (0.903–1.212)	0.549		
Presence of Paracentral VFD (Yes)	0.799 (0.831–5.944)	0.112		
MD (dB)	0.312 (1.110–1.682)	**0.003**	0.333 (1.121–1.736)	**0.003**
Average RNFLT (µm)	0.043 (0.989–1.102)	0.117		
Average CD ratio	− 1.836 (0.004–6.714)	0.336		
Average GCIPLT (µm)	0.048 (0.984–1.119)	0.139		

*CI* confidence interval, *IOP* intraocular pressure, *CCT* central corneal thickness, *VFD* visual field defect, *MD* mean deviation, *RNFLT* retinal nerve fiber layer thickness, *CD* cup disc, *GCIPLT* ganglion cell inner plexiform layer thickness.

Variables with *P* < 0.10 were included in the multivariate analysis.

Factors with statistical significance are shown in bold.

**Figure 3:**
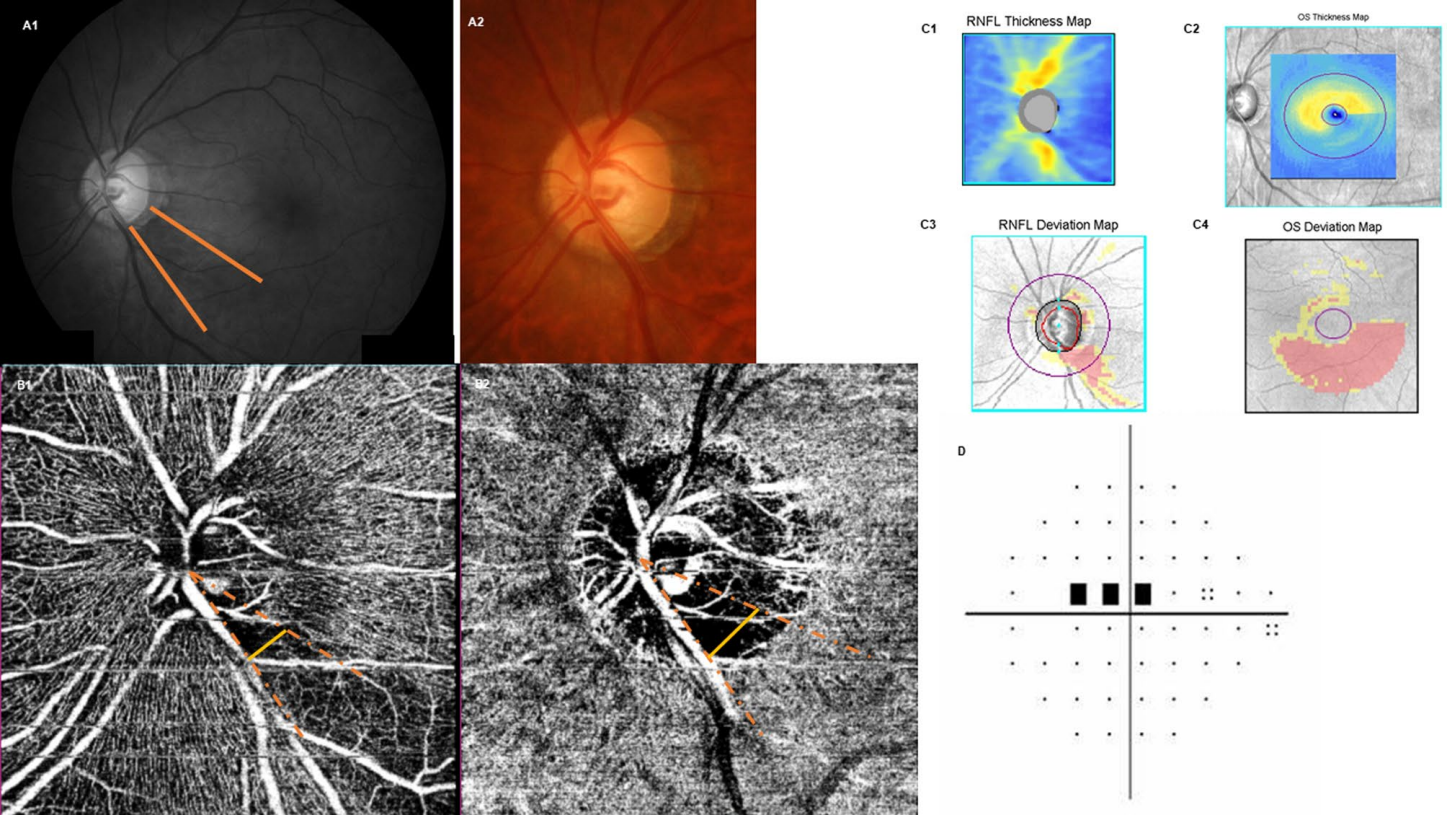
A Representative case of wider localized vessel density defect on the deep layer. A 63-year-old man with normal-tension glaucoma presented with localized inferotemporal retinal nerve fiber layer defect (**A**,**C**) and a paracentral visual field defect (**D**). The signal strength index of optical coherence tomography angiography (OCTA) was 68, and there was the wider vessel density defect in the deep layer map than that of the superficial layer map on OCTA (B-1 and 2, 37.55° and 28.29°, respectively).

**Figure 4:**
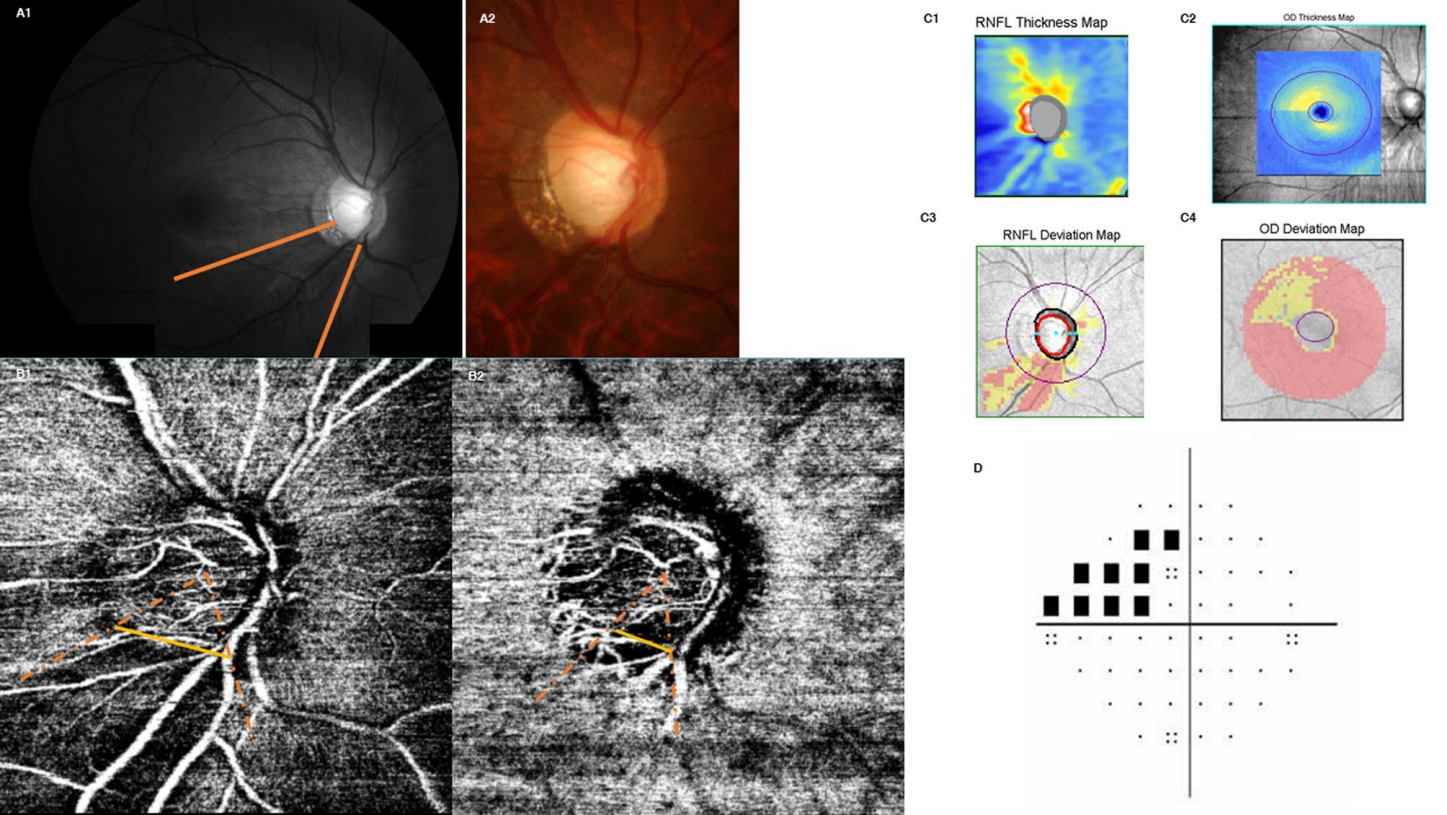
A Representative case of wider localized vessel density defect on the superficial layer. A 45-year-old man had a retinal nerve fiber layer defect at inferotemporal area (**A**,**C**) and correspondent visual field defect was observed at superior (**D**). The image of optical coherence tomography angiography had a good signal strength index, 65, and the measured widths of the vessel density defect were 79.6° for the superficial layer and 58.9° for the deep layer.

Representative cases are shown in Figs. 3 and 4. A 63-year-old man with NTG and hypertension had a paracentral VFD, and a localized inferotemporal RNFL defect (Fig. 3A,C,D). In his OCTA examination, we found a large DVD at the site of inferotemporal RNFL defect, which was wider than the SVD (Fig. 3 B-1,B-2). In the other case, a 45-year-old male NTG patient had a prominent inferotemporal RFNL defect and a superior peripheral VFD (Fig. 4A,C,D). The OCTA exam showed that the width of SVD was noticeably greater than that of DVD (Fig. 4B-1,B-2).

## Discussion

In this study, we found that the width of the SVD was associated with average GCIPLT and PSD, which are one of the parameters reflect glaucomatous damage and status. On the contrary, the patients with wider DVD showed the noticeable relationship with ocular factors including both initial and final IOPs and CCT. Moreover, aging was the significant factor affecting the width of DVD. As to location of VF defect, the significantly higher prevalence of paracentral VF defect in the DVD group was observed. Although baseline average RNFLT and GCILPT were comparable, the severity of the VF defect was worse in the SVD group, compared to the DVD group. These findings could be summarized that superficial VD at the site of localized RNFL defects represents the severity of damage, which results from glaucomatous damage itself. However, deep VD changes at the site of localized RNFL defect are mainly associated with IOP factors and older age, which are regarded to further influence the course of glaucoma^18^. This may explain why deep vessel density is reported to be related to glaucoma progression in recent studies. Therefore, in patients with larger VD in the deep layer on OCTA, closer monitoring according to age and IOP level should be considered.

Previous studies have well shown that a plummet in ocular microvascular circulation is correlated to the status of the disease^10,19^. Although the effect of glaucoma on optic disc microcirculation is still on debate, the OCTA has become a promising implement for diagnosing and monitoring patients. In our study, instead of directly measuring vessel density, we accessed the width of the superficial and the deep VD. Among the OCTA studies, we were not the only group that investigated the width of the VD. According to other studies, angular extent of reduced parapapillary retinal microvasculature showed robust association with RNFL defect^20,21^, presence of disc hemorrhage^21^. Igarashi et al.^22^ analyzed the angle of the radial peripapillary capillaries (RPC) loss, and found that the angle was significantly correlated to the flow density. Additionally, the angle showed markable correlations with PSD, MD, and RNLFT. In our study, average GCIPLT was the most significantly correlated factor, while MD and average RNFLT had mere borderline correlations. These findings suggest that the width of the superficial VD has a potential of a morphological indicator which reflects the changes in glaucoma. The disparity in the correlations with VF indexes in these two studies, however, might be due to the fact that the recruitment of patients in our study was confined to MD >  − 6 dB.

Up to now, most OCTA studies have been reluctant to scrutinize the relationships or changes in the deep layer, of which the majority lies below the INL, and extends to the Bruch’s membrane. This might be due to the fact that the blood flow signal is more likely to be lost in the deep layer and existence of the projection artifacts degrades the quality of the images^13–15^. However, changes in the choroid layer would not be overlooked in light of the fact that insufficient blood supply at the level of lamina cribrosa is regarded as one of the main cause of glaucoma^23,24^. Additionally, the blood supply to prelaminar region of the ONH is mainly dependent on posterior ciliary arteries, which are mainly distributed in peripapillary choroid layer^25^.

Though studies about peripapillary atrophy including choroidal microvascular dropout via OCTA were common^26–28^, to the best of our knowledge, this might be the first study that explored the relationship between the peripapillary deep layer and glaucomatous risk factors. There was a plethora of studies using OCT to evaluate the impact of glaucoma to the peripapillary choroid layer, yet no census has been established. Some reported that peripapillary choroidal thickness does not reflect the severity of glaucoma. Namely, no notable differences in the thickness were observed among controls, glaucoma suspect, POAG, NTG, and PACG^29–31^. Nonetheless, others claimed that the peripapillary choroidal thickness in glaucomatous eyes was obviously thinner than that in eyes of controls or glaucoma suspects^32–34^. Regarding the factors related to the width of deep VD, IOP was the most influential factor. In line with this finding, Ersoz et al.^34^ reported that higher IOP was significantly associated with the peripapillary choroidal thickness, while other parameters, including MD, RNFLT, and lamina and prelaminar thicknesses were not. According to Maul et al.^29^, the significant relationships were found between the peripapillary choroidal thickness and higher IOP, lower diastolic ocular and blood perfusion pressures. Based on the results, he suggested that thick choroid in eyes with higher blood pressure and low IOP was natural with the presence of increased choroidal blood volume with higher diastolic perfusion pressure. In this regard, although we did not measure blood pressure of the patients, we presume that alternations in IOP would affect tremendously to the choroidal circulations in the deep layer by modulating peripapillary choroidal thickness simultaneously. In the present study, baseline IOP with the borderline significance and the IOP measured at OCTA exam with substantial significance were found. In other words, compromised flow in the deep choroidal layer on OCTA around the optic disc due to high IOPs could affect the course of glaucoma and contribute to glaucoma progression.

In terms of CCT, based on the analysis of all eyes, it was not a critical factor for the width of the deep VD. Instead, in eyes with MD <  − 3 dB, thicker CCT had a significant association. Maul et al.^29^ had the same finding, yet the magnitude was more meager, compared to those of other variants. In contrast, instead of CCT, Park et al.^32^ found axial length was the significant determinant with peripapillary choroidal thickness, and other precedent researches^35,36^ suggesting that myopic NTG patients had thinner peripapillary choroidal thickness. However, compared to the patients’ average axial length in Park et al., our patients had longer axial length. Another study reported that CCT was found to be inversely related with macular choroidal thickness, but the multivariate model excluding axial length and anterior chamber depth (ACD) revealed that CCT had no significant correlation with choroidal thickness^30^. Accordingly, further exploration including collecting data of ACD would be necessary for clarification.

With respect to age, the mean of the DVD group was remarkably older than that of the SVD group, and aging was significantly associated with wider DVD. The study, including normal Chinese population, found that the average peripapillary choroidal thickness decreased linearly with age^37^. Hirooka et al.^35^ also showed that age had a correlation with mean peripapillary choroidal thickness in the NTG patients. Histopathologic studies have shown that the vessel density and diameter in the choroid decline with aging^38,39^. Therefore, we hypothesize that the ramification of the compromised choroidal vasculature due to senile effects could be possibly presented as a wider width of the DVD.

Furthermore, we examined the location of the VF defects, and for the DVD group, the prevalence of the paracentral VF defect was significantly higher, compared to the SVD group. Relevance between vascular incompetence and paracentral VF defect in NTG has been analyzed. Specifically, one preceding study suggested that NTG patients with paracentral scotoma were likely to have nocturnal dip and large blood pressure variability^40^. Similarly, there was a finding that medically treated NTG eyes that experienced severe fluctuation in the 24-h ocular perfusion pressure had faster paracentral VF defect progression than eyes with stable pressure^41^. Nevertheless, some studies reported the opposed findings; presence of hemodynamic turbulence has little to do with the location of VF defects^42^, and no significant differences were found between NTG and high tension glaucoma regarding the slopes or depths of the VF defect^43^. Moreover, no difference in systemic and vascular factors between patients with paracentral and peripheral VF defects was found^44,45^. Park et al.^46^ found totally overturned results that eyes with paracentral VFD had distinctive ocular and systemic hemodynamic features. In spite of existence of controversy over the relationship between hemodynamic factors and paracentral VF defect progression, based on our findings, the dominance of paracentral VF defect in the DVD group might imply that damages in choroidal microcirculation contribute to the development of glaucoma ahead of the presence of the glaucomatous changes in the superficial layer.

Our study has several limitations. Firstly, OCTA is a relatively latest implement, compared to OCT. Therefore, it was unavoidable to extract baseline data of OCT and OCTA from different time points. Secondly, there are issues regarding OCTA imaging interpretation, particularly in deep layer. Namely, retinal vessel signals evident on en face, and this hinders researchers to examine deep layer precisely. Nevertheless, recent studies have found that repeatability and reproducibility in measurement were good in superficial layer as well as in deep layer^47,48^. Last, we confined the patients’ pool to early stage of glaucoma. Though it was our aim to examine changes in the inchoate disease, further study may be needed with diverse stage and relatively large number of patients.

In conclusion, in early NTG, various disease parameters showed the distinctive correlations to the respective vascular layer. In other words, the factors showing disease severity, such as average GCIPLT, PSD, were correlated with the width of the superficial VD, while IOP, CCT, and age were significant determinants in the width of the deep VD. Also, the higher frequency of paracentral VF defects in the DVD group was noticeable. Through these findings, vascular circulation not only in superficial layer but also in deep layer would be considered in normal-tension glaucoma patient’s evaluation and management.

## Methods

This study was conducted in a retrospective, observational design, and was approved by the Institutional Review and Ethics Boards (IRB) of Seoul St. Mary’s Hospital, South Korea. (KC20RISI0849). The study also followed all relevant tenets of the Declaration of Helsinki. Informed consent was waived due to the characteristics of retrospective study’s design. The waiver was approved by the IRB of Seoul St. Mary’s Hospital, South Korea who has waived the need of informed consent for this study.

### Study participants and examinations

We collected patient data from July 2011 to July 2020 using the electronic medical record and those patients were referred to the glaucoma clinic at Seoul St. Mary’s Hospital for glaucoma screening. Each participant underwent a comprehensive ophthalmic assessment, including the measurement of best-corrected visual acuity (BCVA), refraction, slit-lamp biomicroscopy, gonioscopy, Goldmann applanation tonometry, central corneal thickness using ultrasound pachymetry (Tomey Corporation, Nagoya, Japan), the determination of axial length (AL) using ocular biometry (IOL Master; Carl Zeiss Meditec, Dublin, CA, USA), dilated stereoscopic examination of the optic disc and fundus, color disc photography, red-free RNFL photography (Canon, Tokyo, Japan), optical coherence tomography (OCT)(Cirrus OCT using software version 6.0; Carl Zeiss Meditec), and Humphrey VF examination (24–2 Swedish Interactive Threshold Algorithm Standard program; Carl Zeiss Meditec) at initial work-up. After the initial visit, the patients were followed-up to the clinic every 6 to 12 months according to their disease severity. Since OCTA (DRI OCT Triton; Topcon) examinations were available from March 1, 2016, all patient could undergo these tests after that point.

Regarding IOP, we set two parameters to analyze their relationship with variables: baseline IOP, which was measured at the first visit of the clinic, and treated IOP was accessed at the last visit regardless of use of glaucoma medication.

The inclusion criteria were: a BCVA of ≥ 20/40, IOP under 21 mmHg, a mean deviation (MD) better than − 6.00 decibels (dB) based on the Hodapp-Anderson-Parish criteria^49^, and presence of a discrete localized RNFL defect either on RNFL photograph or on OCT, OCTA images quality scores greater than 60.

Normal tension glaucoma (NTG) diagnosis was defined by glaucomatous optic disc appearances (such as diffuse or localized rim thinning, a notch in the rim, or a cup-to-disc ratio higher than that of the other eye by > 0.2); IOP of less than 21 mmHg at least two different visits; VF consistent with glaucoma (a cluster of ≥ 3 non-edge points on the pattern deviation plot with a probability of < 5% of the normal population, with one of these points having a probability of < 1%, a pattern standard deviation with *P* < 5%, or a Glaucoma Hemifield Test result outside the normal limits in a consistent pattern on two qualifying VFs), confirmed by two glaucoma specialists (H.Y.-L.P. and C.K.P.); and an open-angle on gonioscopy^50^.

The exclusion criteria were as follows: poor OCT images (due to involuntary saccadic movement, misalignment, or artifacts, and signal strengths < 6); a history of any retinal disease, including diabetic or hypertensive retinopathy or other retinal complications; a history of eye trauma or surgery, including glaucoma incisional surgery or laser treatment; or a history of systemic or neurological diseases possible to affect the VF. If both eyes were eligible, one eye was randomly selected from each patient.

### Optical coherence tomography angiography

The microvasculature of the peripapillary area was imaged via swept- source OCTA device (DRI OCT Triton; Topcon), which uses a laser with a wavelength of 1050 nm and scan speed of 100,000 A scans per second. The OCTA provided en face images through automated layer segmentation around the optic nerve head into 4 layers. Among those, we chose the radial peripapillary capillary (RPC) mode, which estimate a 70 µm thick layer below the internal limiting membrane (ILM), to assess the superficial layer. As for the deep layer, the original choroidal/disc mode was customized for the measurement. Namely, it was intended to measure from 130 µm below the ILM to 390 µm below Bruch’s membrane, however, we manually reset the starting point as 0 µm below inner plexiform layer/inner nucleus layer (IPL/INL) to exclude the changes of the superficial layer.

Images with quality score less than 50 and unclear ocular vascular structures were excluded for further analysis.

### Measurement of the width of vessel density defects using the DRI OCT triton system

**Figure 5:**
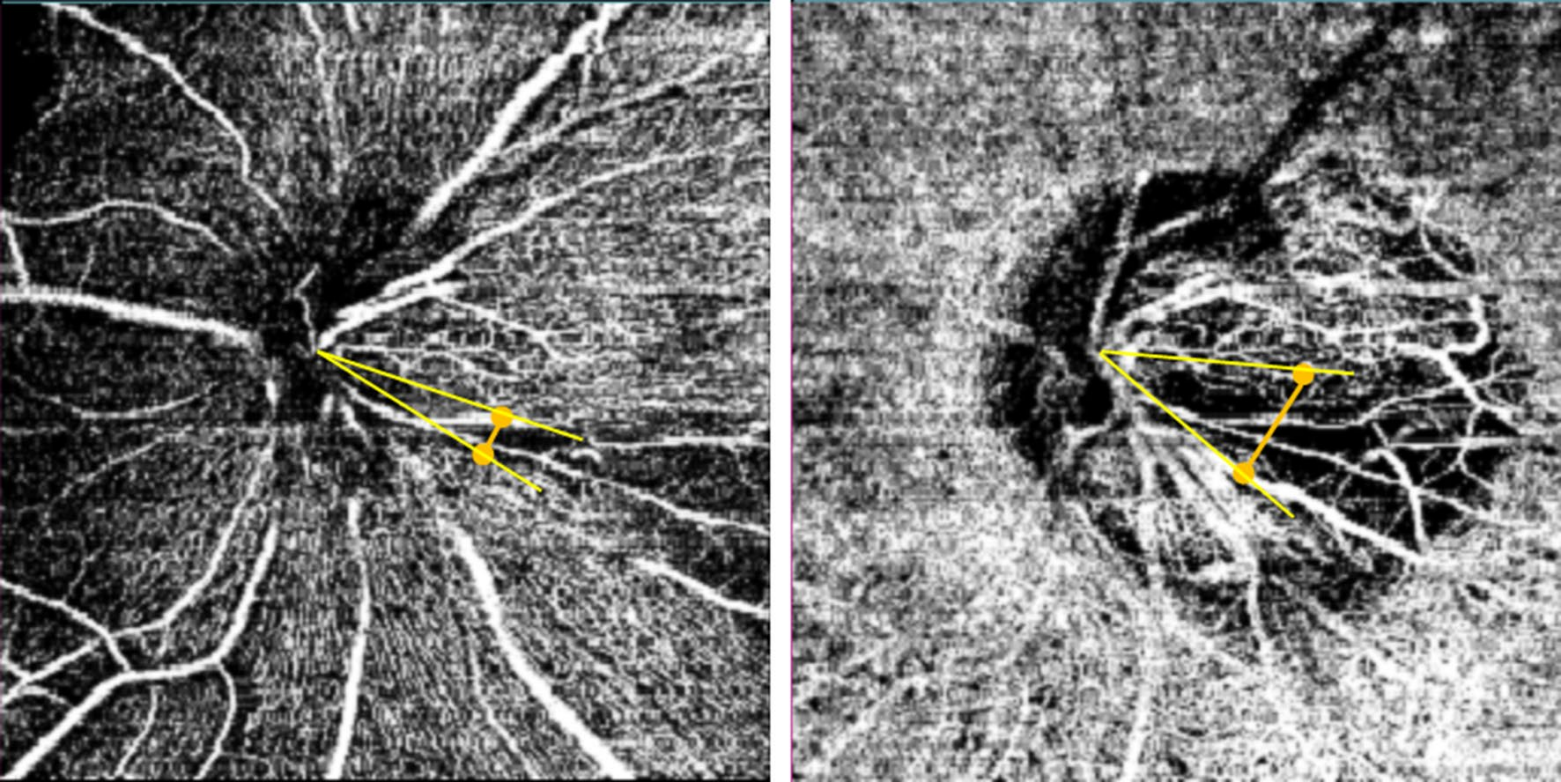
Method for measuring the width of vessel density defect in the image of optical Coherence Tomography Angiography. At the edge of the optic disc, the point where the border of the localized vessel defect (VD) met with the edge was identified. Subsequently, each straight line was drawn to the center of the optic disc. The angle which was generated between two lines was measured, and was defined as the width of the VD.

To assess the width of the VD at the location of the localized RNFL defect, the National Institutes of Health image analysis software (ImageJ version 1.52; available at http://rsb.info.nih.gov/ij/index.html; developed by Wayne Rasband, National Institutes of Health, Bethesda, MD, USA) was adopted. At the edge of the optic disc, the point where the border of the localized VD met with the edge was identified, then straight lines were drawn to the center of the optic disc, respectively. The angle which was generated between two lines was measured, and was defined as the width of the VD (Fig. 5)^22^.

We created two groups: one with wider superficial VD (SVD) group, and the other with wider deep VD (DVD) group. The significant difference between the widths of the VD in each layer was defined as the width variation greater than 10 degree in two layers. Accordingly, if the width of the superficial layer was 10 degree wider than that of the deep layer, the patient was allocated to the SVD group, and vice versa.

### Classification according to the location of the VF defect

Patients were classified as being present or absent of paracentral VF defect, which was defined by presence of one or more significant points in the central 10° with a probability of less than 0.5% on the pattern deviation map.

### Statistical analysis

All statistical analyses were performed using the SPSS statistical package (SPSS, Inc, Chicago, IL, USA) and student’s t-test were adopted to compare the differences between the groups. The chi-square test or Fisher’s exact test was applied to compare frequencies. A *P* value of less than 0.05 was considered statistically significant. Correlation coefficients were calculated to evaluate the relationship between the width of the VD in each layer and ocular or clinical parameters by Pearson correlation analysis. Linear regression analysis was applied to explore meaningful factors affecting the width of the VD. The variables with significance at *P* < 0.10 in univariate analysis were included in the multivariate model. *P* < 0.05 was considered to represent statistical significance.
